# Sphingosine Kinase 1 Regulates the Survival of Breast Cancer Stem Cells and Non-stem Breast Cancer Cells by Suppression of STAT1

**DOI:** 10.3390/cells9040886

**Published:** 2020-04-04

**Authors:** Ling-Wei Hii, Felicia Fei-Lei Chung, Chun Wai Mai, Zong Yang Yee, Hong Hao Chan, Vijay Joseph Raja, Noah Elias Dephoure, Nigel J. Pyne, Susan Pyne, Chee-Onn Leong

**Affiliations:** 1 Centre for Cancer and Stem Cell Research, International Medical University, Bukit Jalil, Kuala Lumpur 57000, Malaysia; lingweihii@imu.edu.my (L.-W.H.); chunwai_mai@imu.edu.my (C.W.M.); yee.zongyang@student.imu.edu.my (Z.Y.Y.); chanhonghao@student.imu.edu.my (H.H.C.); 2 School of Pharmacy, International Medical University, Bukit Jalil, Kuala Lumpur 57000, Malaysia; 3 School of Postgraduate Studies, International Medical University, Bukit Jalil, Kuala Lumpur 57000, Malaysia; 4 Mechanisms of Carcinogenesis Section (MCA), Epigenetics Group (EGE) International Agency for Research on Cancer, World Health Organization, 69372 Lyon, France; chungf@fellows.iarc.fr; 5 Department of Biochemistry, Weill Cornell Medical College, New York, NY 10021, USA; vjr2001@med.cornell.edu (V.J.R.); nod2007@med.cornell.edu (N.E.D.); 6 Strathclyde Institute of Pharmacy and Biomedical Sciences, University of Strathclyde, Glasgow G4 0RE, Scotland, UK; n.j.pyne@strath.ac.uk (N.J.P.); susan.pyne@strath.ac.uk (S.P.)

**Keywords:** cancer stem cells, sphingosine kinase, STAT1, mammospheres, drug synergism, sphingolipids

## Abstract

Cancer stem cells (CSCs) represent rare tumor cell populations capable of self-renewal, differentiation, and tumor initiation and are highly resistant to chemotherapy and radiotherapy. Thus, therapeutic approaches that can effectively target CSCs and tumor cells could be the key to efficient tumor treatment. In this study, we explored the function of SPHK1 in breast CSCs and non-CSCs. We showed that RNAi-mediated knockdown of SPHK1 inhibited cell proliferation and induced apoptosis in both breast CSCs and non-CSCs, while ectopic expression of SPHK1 enhanced breast CSC survival and mammosphere forming efficiency. We identified STAT1 and IFN signaling as key regulatory targets of SPHK1 and demonstrated that an important mechanism by which SPHK1 promotes cancer cell survival is through the suppression of STAT1. We further demonstrated that SPHK1 inhibitors, FTY720 and PF543, synergized with doxorubicin in targeting both breast CSCs and non-CSCs. In conclusion, we provide important evidence that SPHK1 is a key regulator of cell survival and proliferation in breast CSCs and non-CSCs and is an attractive target for the design of future therapies.

## 1. Introduction

Breast cancer stem cells (CSCs) represent a subset of cancer cells with the capabilities of self-renewal and differentiation [1,2]. Although several signaling pathways (such as STAT3 [3,4], Wnt/β-Catenin [5,6,7], Notch [8,9,10,11], Hedgehog [12,13,14,15,16], and NFκB [17,18,19]) have been implicated in regulating the growth and survival of breast CSCs, designing selective CSC-targeted strategies using these pathways remains a challenge as these pathways also share common functional roles in the maintenance of normal stem cells [20,21,22,23].

Sphingosine kinase (SPHK) catalyses the ATP-dependent phosphorylation of sphingosine to form sphingosine 1-phosphate (S1P), which acts as an intracellular second messenger and extracellular ligand for specific receptors [24,25,26]. S1P can be released through specific transporters to act as a ligand for the family of G protein-coupled S1P receptors 1 to 5 (S1P_1_ to S1P_5_) and regulates a wide range of biological effects including transformation and cancer cell survival [24]. S1P levels are tightly regulated by the balance between synthesis by SPHK, reversible conversion to sphingosine by specific S1P phosphatases (SPP1 and SPP2), and degradation by S1P lyase [27]. In contrast to S1P, which is associated with growth and survival, its precursors, sphingosine and ceramide, are associated with cell growth arrest and apoptosis [28]. According to the sphingolipid rheostat model, the balance between these interconvertible sphingolipids, ceramide, sphingosine, and S1P, regulates cellular growth and survival in response to cellular and environmental stimuli [29,30,31,32,33]. Thus, SPHK is a critical regulator of this rheostat, as it produces the pro-growth and anti-apoptotic S1P and also reduces levels of pro-apoptotic ceramide and sphingosine [26,30,34,35,36,37]. Thus, the inhibition of SPHK is likely to have an anti-cancer effect by producing apoptotic ceramide/sphingosine.

There are two isoforms of sphingosine kinase called SPHK1 and SPHK2. Several studies have indicated that increased SPHK1 activity promotes cancer cell growth, metastasis, and inhibits apoptosis [24,25,26,38,39,40,41]. Indeed, high expression of SPHK1 in tumors is associated with worse prognosis and overall outcomes in breast cancer patients [41,42,43,44,45,46,47,48]. In addition, overexpression of SPHK1 in breast cancer cells was reported to increase breast CSCs and the tumorigenicity of tumors in nude mice via S1P binding to S1P_3_ and down-stream stimulation of Notch and p38 MAPK signaling [49]. Furthermore, benzyl butyl phthalate, a carcinogen that has been shown to induce SPHK1 expression through activation of the aryl hydrocarbon receptor (AhR), was recently shown to enhance the formation of metastasis-initiating breast CSCs, suggesting a role of SPHK1 in breast CSCs [50].

Using a kinome-wide shRNA library screen, we previously identified that SPHK1 is required for breast cancer cell survival [51]. However, whether SPHK1 is required for the survival of breast CSCs remains unknown. Hence, this study sought to investigate whether SPHK1 regulates the survival of breast CSCs, the underlying mechanism of this protection, and whether there are any substantive differences with its role in non-CSCs. In this regard, we demonstrate herein that SPHK1 expression is increased in breast CSCs compared with non-CSCs and is involved in regulating the survival of breast CSCs and non-CSCs through repression of the tumor suppressor function of STAT1. Importantly, selective inhibition of SPHK1 enhances doxorubicin sensitivity in breast CSCs and non-CSCs. Overall, our results implicate SPHK1 as a potential target for the treatment of refractory breast cancers by targeting both breast CSCs and non-CSCs.

## 2. Materials and Methods

### 2.1. Cell Lines and Cell Culture

Human breast cancer cell lines, MCF-7, SKBR3, MDA-MB-468, and HCC38, were obtained from American Type Culture Collection (ATCC, Manassas, VA, USA). Parental cells were cultured in RPMI-1640 media containing 10% fetal bovine serum, 100IU/mL penicillin, and 100 µg/mL streptomycin (Sigma-Aldrich, USA). To establish the breast CSC culture, parental breast cancer cells were seeded at a density of 3 × 10^4^ cells/well in ultra-low attachment 6 well plates (Corning Inc., Corning, NY, USA) in 2mL of MammoCult media (Stem Cell Technologies, Vancouver, Canada) supplemented with 4 µg/mL heparin (Stem Cell Technologies, Canada), 0.48 µg/mL hydrocortisone, 100IU/mL penicillin, and 100µg/mL streptomycin (Sigma-Aldrich, St. Louis, MO, USA), as described previously [11,52,53,54,55,56]. After 5 days of culturing, the resulting mammospheres were centrifugated at 1500 rpm, and the cell pellet was trypsinized with 1× Trypsin EDTA, followed by CD44-APC and CD24-PE (BD Biosciences, San Jose, CA, USA) flow cytometry to confirm the enrichment of CD44^+^/CD24^-^ CSCs [52]. Mammosphere forming efficiency (MFE) was calculated as the ratio of the number of mammospheres divided by the initial number of cells plated.

### 2.2. Immunoblotting

Total proteins were extracted using ice-cold lysis buffer (1% NP-40, 1mM DTT, phosphatase inhibitor and protease inhibitor cocktail in PBS) followed by immunoblotting [57,58]. Primary antibodies against SPHK2, pSTAT1 (Y701), STAT1, IRF9, pSTAT2 (Y690), and STAT2 were purchased from Cell Signalling Technologies, USA. Anti-phosphorylated SPHK1 (S225) was obtained from ThermoFisher Scientific, Waltham, Massachusetts, USA; anti-SPHK1 from Abcam, USA; and anti-GAPDH from Santa Cruz Biotechnology, Santa Cruz, CA, USA. All images were captured using the ChemiDocTM XRS+ molecular imager (Bio-Rad Laboratories, Hercules, CA, USA). 

### 2.3. S1P ELISA 

Sphingosine 1 phosphate (S1P) levels were determined using the S1P ELISA kit (MyBioSource, San Diego, CA, USA) according to the manufacturer’s instructions. Absorbance readings were recorded at 450nm using a SpectraMax^®^ M3 multi-mode microplate reader (Molecular Devices, San Jose, CA, USA). 

### 2.4. Lentivirus Production and Transduction 

The pLKO.1 lentiviral vector (Vec), non-targeting shRNA (NS), and shRNA constructs targeting SPHK1 and STAT1 were purchased from Sigma-Aldrich and Dharmacon, respectively. The target sequences are shown in Table S4. Lentivirus production and transduction were performed as described previously [51,59]. Briefly, packaging plasmids (psPAX2; Addgene plasmid: 12260) and envelope plasmids (pMD2.G; Addgene plasmid: 12259) were co-transfected into HEK-293T using CalPhos Transfection Kits (Clontech, Mountain View, CA, USA) to produce a high-titer lentiviral stock. The lentiviral particles were supplemented with 7.5 μg/mL polybrene (Sigma-Aldrich, St. Louis, MO, USA) and transduced to adherent cells or mammospheres. The stable pools of cells were generated using an shRNA construct targeting STAT1, and puromycin (Sigma-Aldrich, St. Louis, MO, USA) selection. 

### 2.5. Cell Proliferation Assay

Cell viability was quantified using CellTiter 96^®^ AQueous One Solution Cell Proliferation Assay (Promega, Madison, WI, USA; generally referred to as the MTS cell viability assay) [60]. Both attached cells and mammospheres were seeded in 96 well plates overnight before being exposed to treatment for 72 h. CellTiter 96^®^ AQueous One Solution Reagent was added to each well and further incubated for 4 h. The MTS tetrazolium compound was bioreduced by viable attached cells or mammospheres to form a stable colored formazan product. The absorbance of the resulting formazan was measured using a TECAN Infinite F200 96 well plate reader (Tecan Group Ltd., Männedorf, Switzerland) at 540 nm. 

### 2.6. Caspase Activation Assay

The caspase 3/7, 8, and 9 activities were measured using the Caspase-Glo 3/7, Caspase-Glo 8, and Caspase-Glo 9 Assay kits (Promega, Madison, WI, USA), respectively, according to the manufacturer’s instructions. 

### 2.7. Proteomic Profiling 

The SPHK1 plasmid was ectopically expressed in HEK293 cells followed by proteomic profiling using LC-MS/MS analysis (Supplemental Methods). Processing and protein identification were conducted as described previously [61]. Proteins were defined as differentially expressed when expression differed by >2.0 fold between control and SPHK1-overexpressing cells and were significant when tested with the Student’s *t*-test with a cut-off point of *p* < 0.05. Differentially expressed genes were mapped to known molecular pathways using DAVID Functional Annotation Bioinformatics Tool v6.8 (https://david.ncifcrf.gov/).

### 2.8. ISRE and GAS Luciferase Reporter Assay

SPHK1 shRNAs were co-transfected with an IFN-stimulated response element (ISRE) or gamma-activated sequences (GAS) luciferase reporter (Qiagen, Germantown, MD, USA) using X-tremeGENE HP DNA transfection reagent (Roche Diagnostics, Indianapolis, IN, USA). ISRE or GAS luciferase activities were determined using a SpectraMax^®^ M3 multi-mode microplate reader (Molecular Devices, San Jose, CA, USA) at 48 h after transfection.

### 2.9. Apoptosis Assay

Both floating and attached cells were collected and stained with the PE-Annexin V Apoptosis Detection Kit (BD Biosciences, San Jose, CA, USA), according to the manufacturer’s instructions. Results were recorded using an FACSCalibur flow cytometer (BD Biosciences, USA) and analyzed using CellQuest Pro Software (BD Biosciences, San Jose, CA, USA). 

## 3. Results

### 3.1. The SPHK1-S1P Axis Is Hyperactivated in Breast CSCs

Both SPHK isoforms, SPHK1 and SPHK2, are reported to be involved in regulating oncogenesis in human cancers [62,63]. To investigate whether the SPHK-S1P axis is altered in breast CSCs, we evaluated the basal expression levels of SPHK1, phosphorylated SPHK1, and SPHK2 in a panel of breast CSCs derived from MCF-7, SKBR3, MDA-MB-468, and HCC38 breast cancer cells. Of note, the breast CSCs enriched from the breast cancer cell lines have been previously shown to contain functional cancer stem cells with high CD44 and low CD24 expression and retain high tumorigenic activity when injected into the mammary fat pad of SCID mice [52,53,54,55,56].

**Figure 1:**
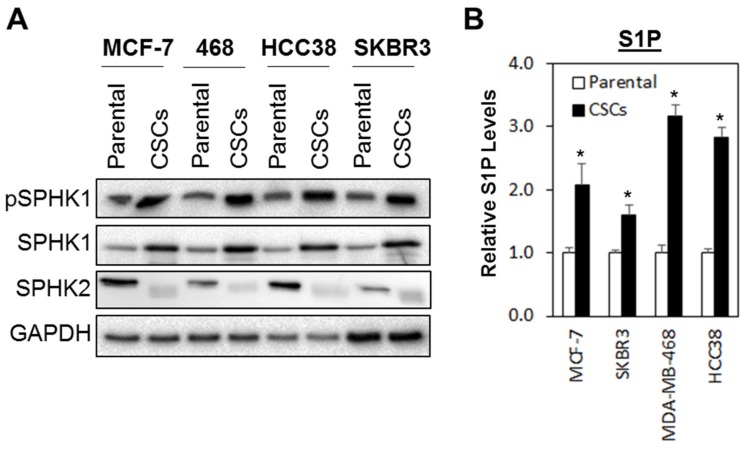
SPHK1 protein and S1P secretion are increased in breast cancer stem cells (CSCs) compared to adherent parental cells. (**A**) SPHK1 and phosphorylated SPHK1 protein expression was upregulated, while SPHK2 expression was downregulated in CSCs derived from MCF-7, SKBR3, HCC38, and MDA-MB-468 breast cancer cells. (**B**) S1P secretion was increased in CSC cultures compared to their respective parental cells. Bars represent the means ± s.d. of three independent experiments. Asterisks (*) indicate statistical significance compared with parental cells (*p* < 0.01, Student’s *t*-test).

As shown in Figure 1A, phosphorylated SPHK1 and total SPHK1 were consistently upregulated in all the breast CSCs tested as compared with the parental cells, while the inverse was observed for SPHK2, where higher levels of expression were detected in the parental cells compared with breast CSCs. These expression patterns, however, were not observed at the mRNA levels, suggesting that the upregulation of SPHK1 and downregulation of SPHK2 in breast CSCs are independent of transcription activation and might be regulated at the post-transcriptional level, possibly at the level of protein stability (Figure S1).

Similarly, secreted S1P was also significantly elevated in breast CSCs compared to the parental cells (Figure 1B), suggesting that the upregulation of SPHK1 alone is sufficient to compensate for the downregulation of SPHK2 in the production S1P in breast CSCs.

### 3.2. Depletion of SPHK1 Inhibits Breast CSCs’ and Non-CSCs’ Survival and Proliferation

**Figure 2:**
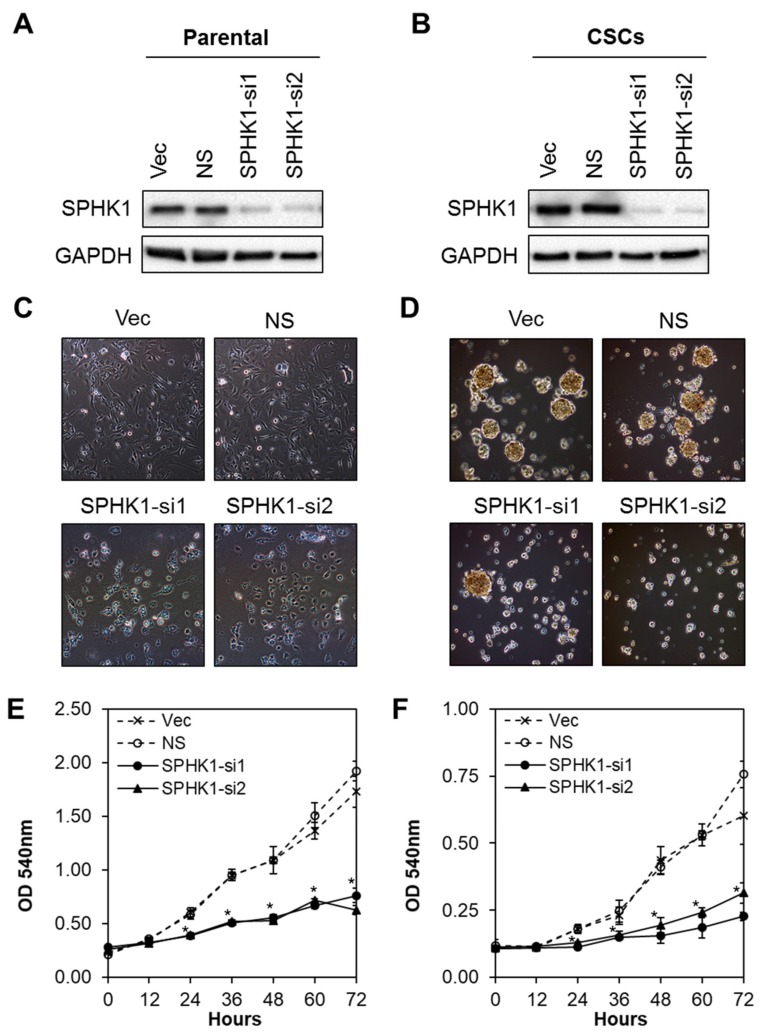
Depletion of SPHK1 inhibits HCC38 CSC and non-CSC proliferation. (**A**,**B**) Efficient depletion of SPHK1 expression was achieved using two independent lentiviral shRNA constructs in non-CSC and CSC cultures. Vector (Vec) and non-silencing (NS) controls were included for accurate assessments of knockdown efficiency; GAPDH was used as a loading control. (**C**,**D**) Morphological changes were observed following knockdown of SPHK1 in non-CSC and CSC cultures. (**E**,**F**) Cell proliferation between 0 and 72 h was significantly reduced in SPHK1-depleted non-CSC and CSC cultures. Points represent the means ± s.d. of three independent experiments. Asterisks (*) indicate statistical significance compared with non-silencing (NS) control cells (*p* < 0.01, Student’s *t*-test).

**Figure 3:**
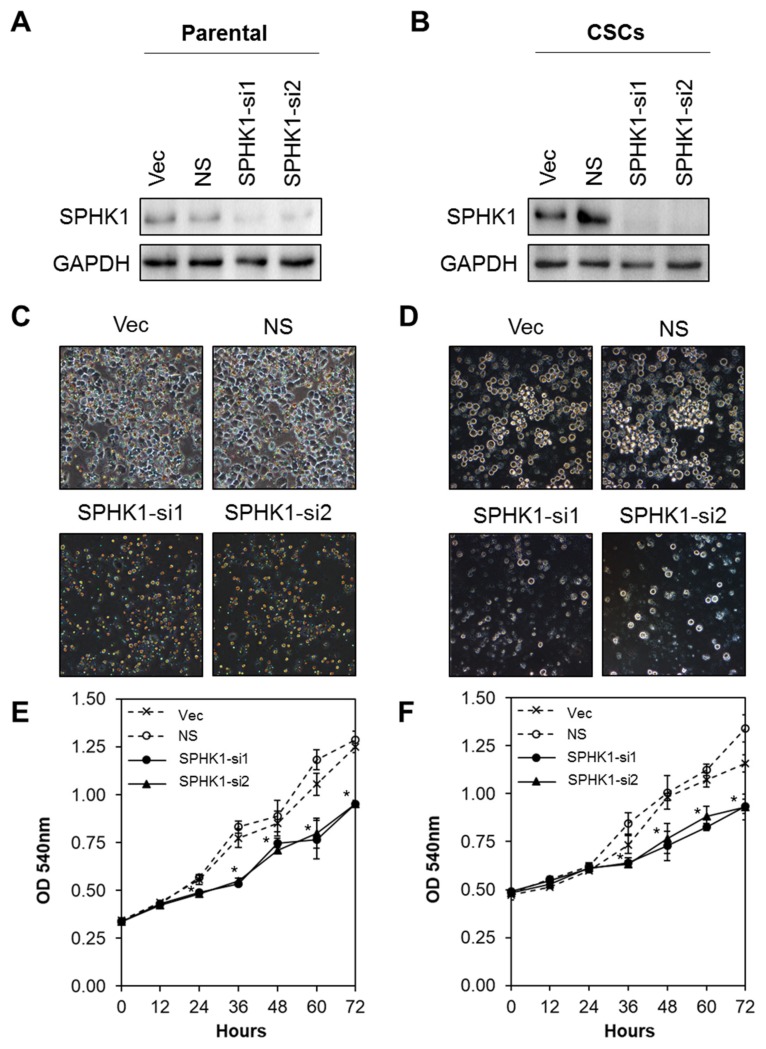
Depletion of SPHK1 inhibits MDA-MB-468 CSCs’ and non-CSCs’ proliferation. (**A**,**B**) Efficient depletion of SPHK1 expression was achieved using two independent lentiviral shRNA constructs in non-CSC and CSC cultures. Vector (Vec) and non-silencing (NS) controls were included for accurate assessments of knockdown efficiency; GAPDH was used as a loading control. (**C**,**D**) Morphological changes were observed following knockdown of SPHK1 in non-CSC and CSC cultures. (**E**,**F**) Cell proliferation between 0 and 72 h was significantly reduced in SPHK1-depleted non-CSC and CSC cultures. Points represent the means ± s.d. of three independent experiments. Asterisks (*) indicate statistical significance compared with non-silencing (NS) control cells (*p* < 0.01, Student’s *t*-test).

**Figure 4:**
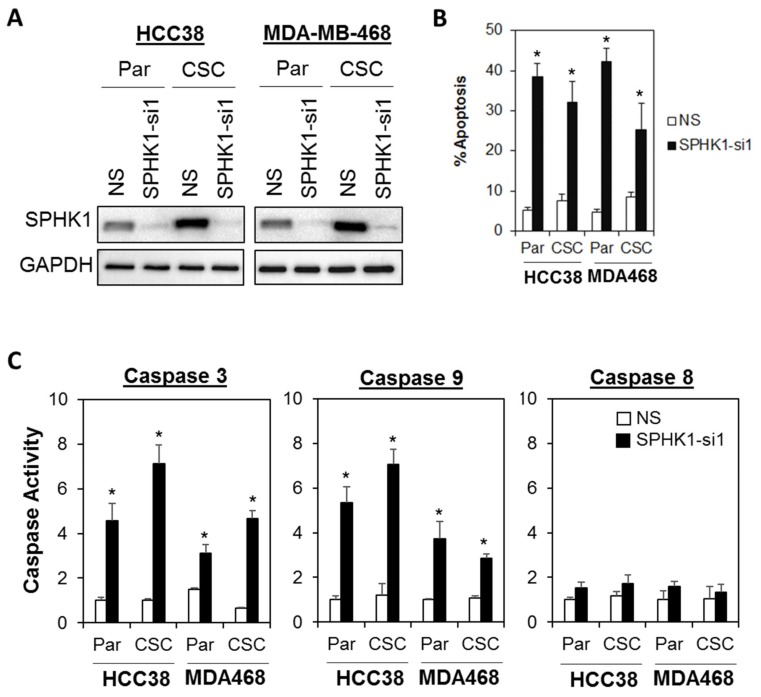
Depletion of SPHK1 induces cell apoptosis in breast CSC and non-CSC cultures. (**A**) Efficient depletion of SPHK1 expression was achieved using a single lentiviral shRNA construct in HCC38 and MDA-MB-468 adherent parental (Par) and CSC cultures. Non-silencing (NS) controls were included for accurate assessments of knockdown efficiency; GAPDH was detected as a loading control. (**B**) The proportion of cells undergoing apoptosis was assessed by Annexin V/7-AAD flow cytometry at 72 h following SPHK1 knockdown. Note the significant induction of apoptosis in transduced parental and CSCs. (**C**) Depletion of SPHK1 expression activates caspase 3/7 and 9, but not caspase 8. Both parental and CSCs were transduced with lentiviral shRNA targeting endogenous SPHK1. Caspase activities were determined using the CaspaseGlo assay at 72 h after transduction. Bars represent the means ± s.d. of three independent experiments. Asterisks (*) indicate statistical significance compared with non-silencing (NS) control cells (*p* < 0.01, Student’s *t*-test).

Next, we evaluated whether SPHK1 was involved in regulating the survival and/or proliferation of breast CSCs and non-CSCs. We knocked down the expression of endogenous SPHK1 in HCC38 and MDA-MB-468 parental and CSCs using two independent lentiviral shRNAs followed by evaluation of cell proliferation using the MTS assay. As shown in Figure 2A,B and Figure 3A,B, efficient knockdown of SPHK1 expression was demonstrated at the protein level for both shRNA constructs (SPHK1-si1 and SPHK1-si2) in HCC38 and MDA-MB-468 parental and CSCs. SPHK1 depletion was accompanied by significant reductions in cell proliferation in both the parental and CSCs of HCC38 and MDA-MB-468 (Figure 2C–F and Figure 3C–F). The effects of SPHK1 depletion on cell survival were assessed using Annexin V/7-AAD flow cytometry. We observed significant apoptosis in SPHK1-depleted parental and CSCs (*p* < 0.01, Student’s *t*-test) corroborated by a significant induction of caspase 3/7 and 9 activities (*p* < 0.01, Student’s *t*-test), but not caspase 8 activity (Figure 4A–C and Figure S2). These results indicated that SPHK1 is required for breast CSCs’ and non-CSCs’ survival and proliferation. 

### 3.3. Ectopic Expression of SPHK1 Enhances Breast CSCs’ Survival and Mammosphere Forming Efficiency, but not Cell Fate Transition

**Figure 5:**
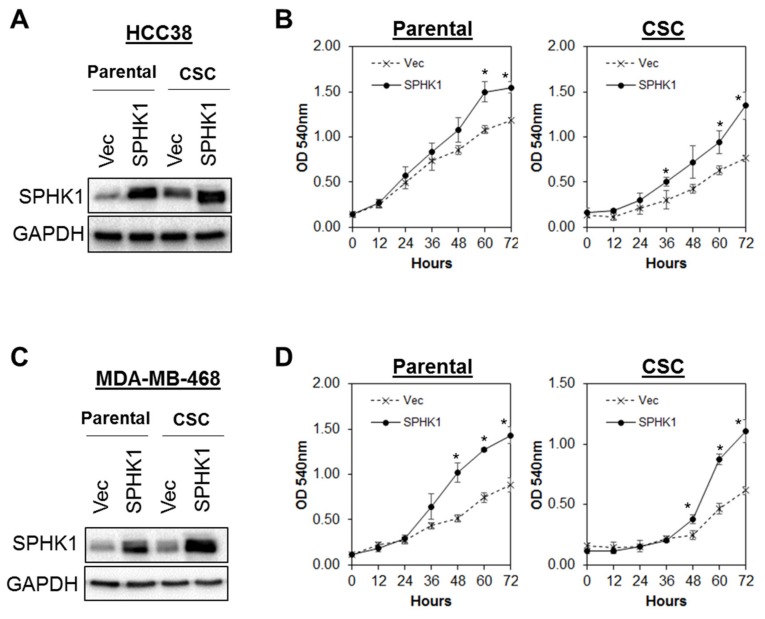
Overexpression of SPHK1 promotes cell proliferation in breast CSCs and non-CSCs. SPHK1 was overexpressed in (**A**,**B**) HCC38 and (**C**,**D**) MDA-MB-468 parental and CSCs. Cells’ proliferation was determined using the MTS assay. Note the increases in cell proliferation in both parental and CSCs following SPHK1 overexpression. Points represent the means ± s.d. of three independent experiments. Asterisks (*) indicate statistical significance compared with vector (Vec) control cells (*p* < 0.01, Student’s *t*-test).

**Figure 6:**
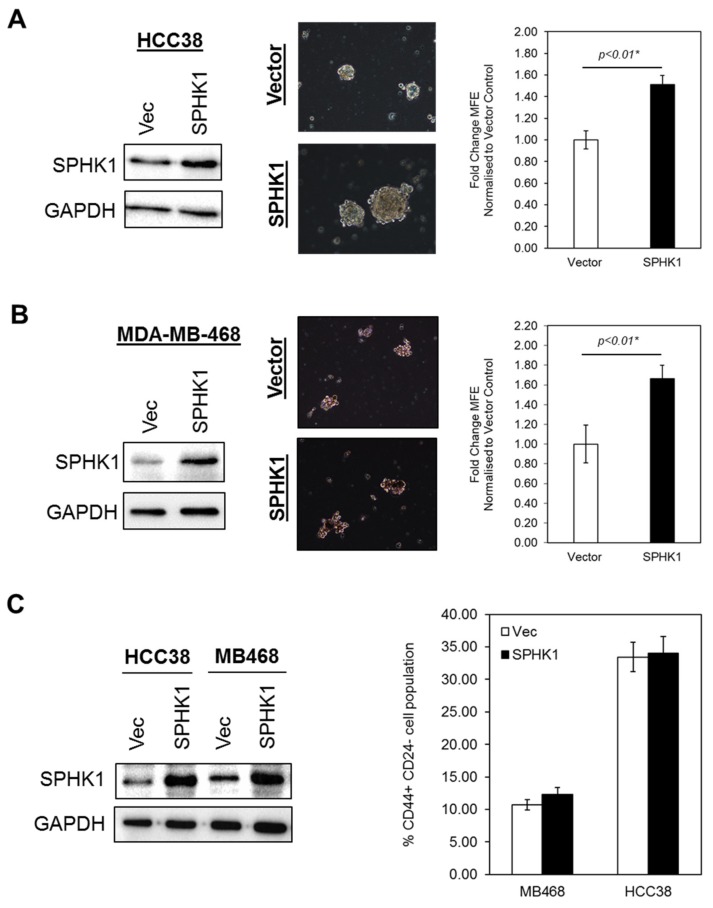
Overexpression of SPHK1 promotes mammosphere formation, but does not affect the proportion of stem-like cells in parental cultures. (**A**,**B**) SPHK1 was overexpressed in HCC38 and MDA-MB-468 prior to mammosphere formation assays. The number of mammospheres was counted for each subline and the mammosphere forming efficiency (MFE) determined as the proportion of mammosphere-forming cells relative to the number of single cells seeded. Results are expressed as normalized ratios against the MFE of the vector control cells. Representative phase contrast microscope images are shown. (**C**) Overexpression of SPHK1 in parental HCC38 and MDA-MB-468 has no effect on the proportion of CD44^+^/CD24^low/-^ cells. Bars represent the means ± s.d. of three independent experiments. Asterisks (*) indicate statistical significance compared with vector (Vec) control cells (*p* < 0.01, Student’s *t*-test).

Given the fact that SPHK1 has been shown to regulate breast CSCs expansion and tumorigenicity [49], we tested whether overexpression of SPHK1 enhances mammosphere formation and increases the population of CSCs. To test this hypothesis, we generated a panel of isogenic cell lines with ectopic expression of SPHK1 in HCC38 and MDA-MB-468 cells. Our results showed that overexpression of SPHK1 induced significant cell proliferation and enhanced mammosphere forming efficiency (MFE) in both parental and CSCs of HCC38 and MDA-MB-468 (Figure 5 and Figure 6A,B; *p* < 0.01, Student’s *t*-test). Interestingly, no significant changes in the CD44^+^/CD24^-/low^ cell population in the parental cells overexpressing SPHK1 were observed, suggesting that SPHK1 promotes breast CSC survival and mammosphere formation, but does not drive transition [64]. 

### 3.4. Proteomic Profiling Identifies STAT1 as the Major Target Regulated by SPHK1

**Figure 7:**
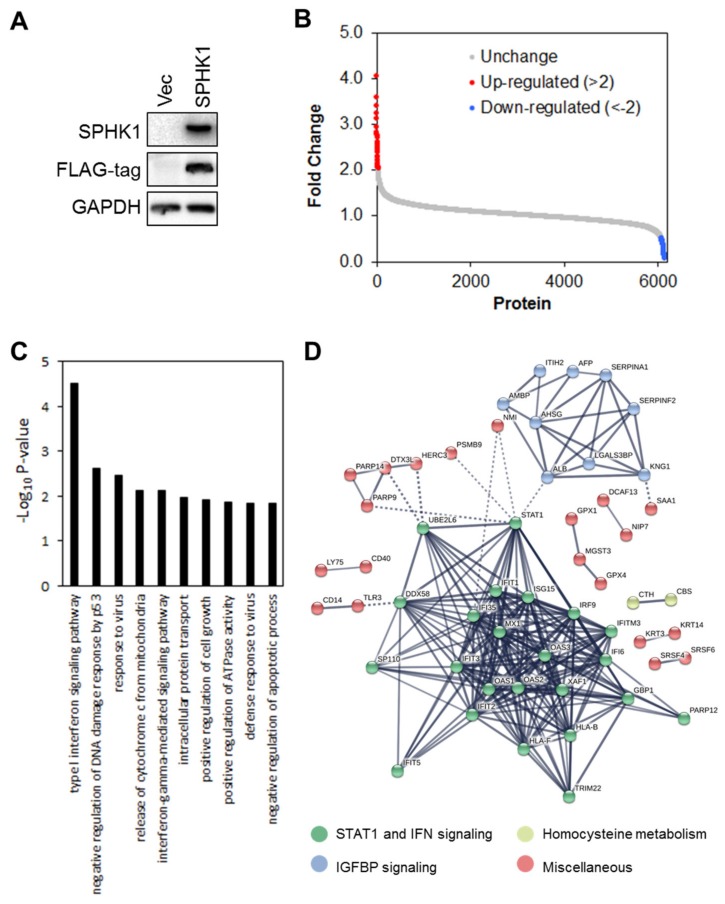
Multidimensional protein identification technology (MudPIT) identifies novel targets of SPHK1. (**A**) SPHK1 was overexpressed in HEK-293 cells. Protein lysates were isolated and subjected to MudPIT analyses. As evident from the FLAG-tag immunoblot, virtually all of the detected SPHK1 is exogenously expressed. (**B**) A total of 34 proteins were upregulated (more than two-fold,) while 74 proteins were downregulated. (**C**) The top 10 most enriched GO terms in proteins deregulated by SPHK1 overexpression, determined by DAVID enrichment analysis. Type I and II interferon signaling are two of the most significantly perturbed pathways in SPHK1-overexpressing cells. (**D**) Protein-protein interaction network of SPHK1 target proteins. Networks were generated with STRING using a confidence threshold of 0.7.

We next investigated the mechanism by which SPHK1 regulates the proliferation and survival of breast CSCs and non-CSCs to establish which signaling networks are functional. To define potential signaling networks regulated by SPHK1, we conducted a global proteomic analysis using multidimensional protein identification technology (MudPIT) in ectopic SPHK1 overexpressing HEK-293 cells (Figure 7A). A total of 35 proteins were found to be upregulated (fold change > 2), while 77 were downregulated (Figure 7B and Tables S1 and S2).

Further functional enrichment analysis using DAVID Functional Annotation Tools revealed that expression of SPHK1 is significantly associated with type I and II interferon signaling pathways (Figure 7C and Table S3). Indeed, unsupervised clustering of differentially regulated proteins in HEK293 cells overexpressing SPHK1 enabled identification of three major clusters of proteins. These were: (1) STAT1 and IFN signaling, (2) IGFBP signaling, and (3) homocysteine metabolism. We focused our attention on STAT1 and IFN signaling in breast CSC and parental cells because STAT1 is a negative regulator of proliferation and cell survival [65], and this could account for the function of SPHK1 in promoting these cell responses reported herein.

### 3.5. Depletion of SPHK1 Induces STAT1 and Activates Type I and II Interferon Signalling

**Figure 8:**
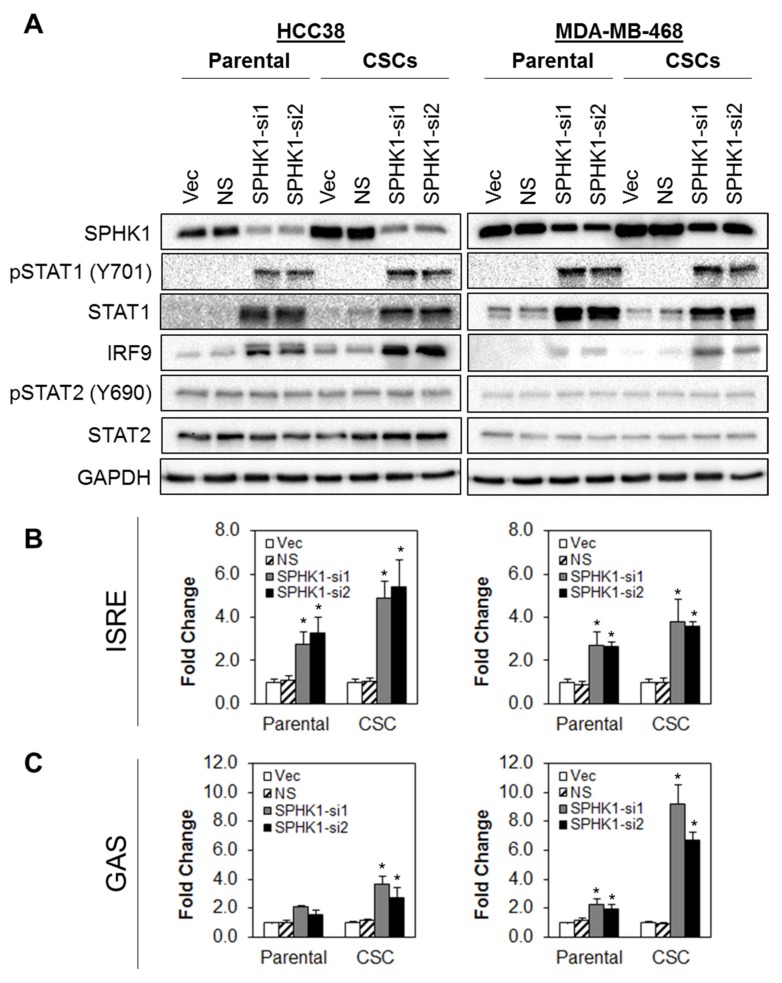
Depletion of SPHK1 induces increased expression of STAT1 and activates type I and type II interferon signaling. (**A**) Depletion of SPHK1 expression in parental and CSCs of HCC38 and MDA-MB-468 induced expression of total STAT1, pSTAT1, and IRF9. Expression of total STAT2 and pSTAT2 was unchanged. (**B**,**C**) Knockdown of SPHK1 induced type I and II interferon transcriptional activities. HCC38 and MDA-MB-468 parental and CSCs were co-transfected with the SPHK1 shRNA IFN-stimulated response element (ISRE) or gamma-activated sequences (GAS) luciferase reporter constructs. Luciferase activities were determined at 48 h after transfection. Bars represent the means ± s.d. of three independent experiments. Asterisks (*) indicate statistical significance compared with non-silencing (NS) control cells (*p* < 0.01, Student’s *t*-test).

To dissect the interactions between SPHK1 and IFN signaling and to validate the MudPIT findings, the effect of SPHK1 depletion on protein expression of STAT1, pSTAT1 (V701), STAT2, pSTAT2 (Y690), and IRF9 was determined in parental and CSCs derived from HCC38 and MDA-MB-468 cells (Figure 8A). Depletion of SPHK1 resulted in upregulation of STAT1, pSTAT1, and IRF9 in both parental and CSCs, while STAT2 and pSTAT2 expression levels remained unchanged. 

Since STAT1 has been shown to play a pivotal role in type I and type II interferon (IFN) signaling and possess tumor suppressor activities, particularly in mammary tumor formation [66,67,68], we hypothesized that one mechanism by which SPHK1 might promote breast CSCs’ and non-CSCs’ survival is through suppression of STAT1. 

Of note, upon activation, phosphorylated STAT1 is known to interact with STAT2 and IRF9 to form the interferon-stimulated gene factor 3 (ISGF3) complex. ISGF3, in turn, binds to IFN-stimulated response element (ISRE) sequences to activate classical STAT1 target genes [69]. Alternatively, STAT1 might also form homodimers that bind to the gamma-activated sequences (GASs) to induce pro-inflammatory genes’ expression [69]. 

To test whether the knockdown of SPHK1 activates STAT1-dependent transcription through ISRE or GAS, we co-transfected SPHK1 shRNA together with an ISRE or GAS luciferase reporter into parental and CSCs derived from HCC38 and MDA-MB468 cells. As shown in Figure 8B, depletion of SPHK1 led to significant activation of ISRE-dependent reporter gene transcription in parental and CSC cultures in both cell lines. Similarly, SPHK1 depletion also activated GAS-dependent luciferase expression in HCC38 CSCs and in MDA-MB-468 parental and CSCs, while a modest increase in GAS-dependent reporter activity was observed in HCC38 parental cells. 

### 3.6. Apoptosis Induced by SPHK1 Depletion Is Mediated through STAT1

**Figure 9:**
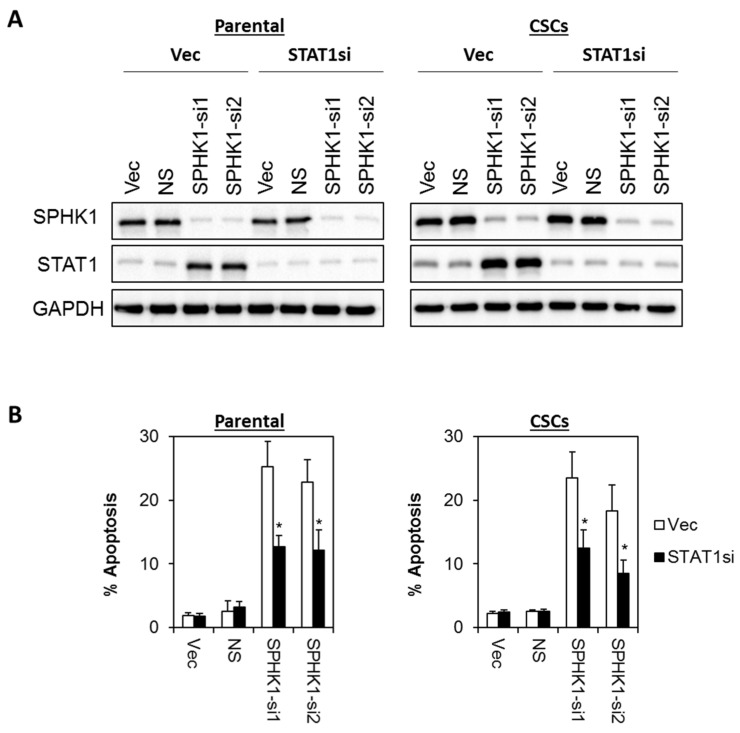
Apoptosis induced by SPHK1 depletion in HCC38 parental and CSCs is dependent on STAT1 function. (**A**) A stable pool of STAT1 depleted cells was generated by transduction of lentiviral shRNA targeting STAT1, followed by brief puromycin selection. Cells were then transfected with SPHK1 shRNAs. Immunoblots were performed at 72 h after SPHK1 knockdown. (**B**) Depletion of STAT1 rescued SPHK1 knockdown induced apoptosis. Apoptosis was quantitated using Annexin V/7-AAD flow cytometry at 72 h after SPHK1 knockdown. Bars represent the means ± s.d. of three independent experiments. Asterisks (*) indicate statistical significance compared with non-silencing (NS) control cells (*p* < 0.01, Student’s *t*-test).

**Figure 10:**
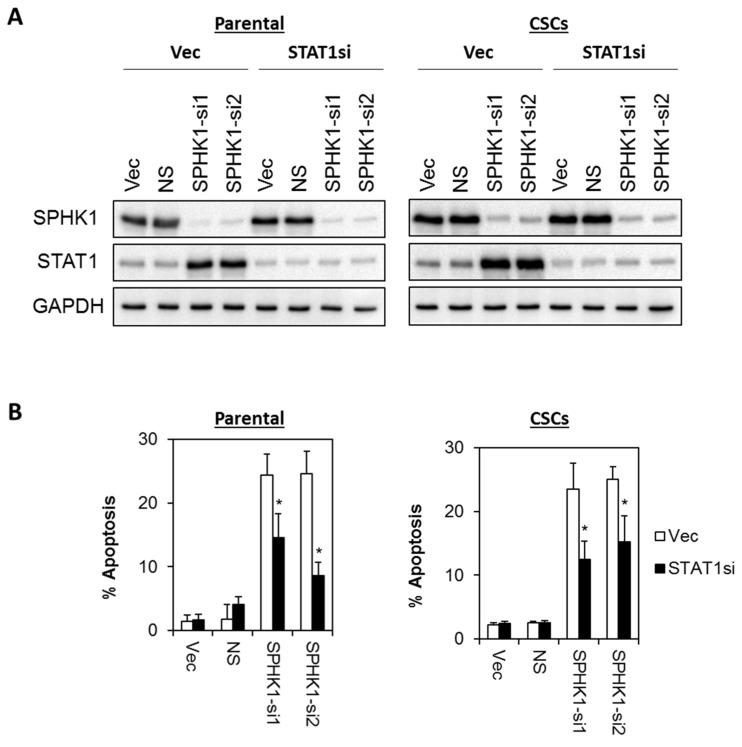
Apoptosis induced by SPHK1 depletion in MDA-MB-468 parental and CSC is dependent on STAT1 function. (**A**) A stable pool of STAT1 depleted cells was generated by transduction of lentiviral shRNA targeting STAT1 followed by brief puromycin selection. Cells were then transfected with SPHK1 shRNAs. Immunoblots were performed at 72 h after SPHK1 knockdown. (**B**) Depletion of STAT1 rescued SPHK1 knockdown induced apoptosis. Apoptosis was quantitated using Annexin V/7-AAD flow cytometry at 72 h after SPHK1 knockdown. Bars represent the means ± s.d. of three independent experiments. Asterisks (*) indicate statistical significance compared with non-silencing (NS) control cells (*p* < 0.01, Student’s *t*-test).

Next, we investigated whether SPHK1 reduces the tumor suppressive function of STAT1. Thus, we evaluated the apoptotic effects of SPHK1 depletion in a stable pool of STAT1 knockdown cells using two independent STAT1 shRNAs. As shown in Figure 9 and Figure 10, depletion of STAT1 significantly rescued the apoptosis induced by the knockdown of SPHK1 expression in both parental and CSCs of HCC38 and MDA-MB-468, suggesting that SPHK1 functions to promote the survival of breast CSCs and non-CSCs via a STAT1-dependent mechanism.

### 3.7. SPHK1 Inhibitors Enhance Doxorubicin Sensitivity in Basal-Like Breast CSCs and Non-CSCs.

Since our results showed that SPHK1 plays functional roles in regulating the survival of breast CSCs and non-CSCs and given that recent studies have highlighted the potential of utilizing agents that manipulate sphingolipid metabolism to augment the chemotherapeutic efficacy in hematological malignancies [70], we sought to investigate whether inhibitors targeting SPHK1 and the sphingolipid rheostat might exert anti-proliferative effects in breast CSCs and non-CSCs.

A total of four inhibitors targeting SPHK were used and tested in combination with doxorubicin. FTY720 inhibits SPHK1, and its phosphorylated equivalent modulates S1P receptors [71,72]; PF543 is a nM potent selective inhibitor of SPHK1 [73]; SK1-II acts as dual SPHK1/SPHK2 inhibitor; and ABC294640 is a selective μM potent SPHK2 inhibitor [74], but in androgen independent prostate cancer cells can also induce the proteasomal degradation of SPHK1 [75]. 

**Figure 11:**
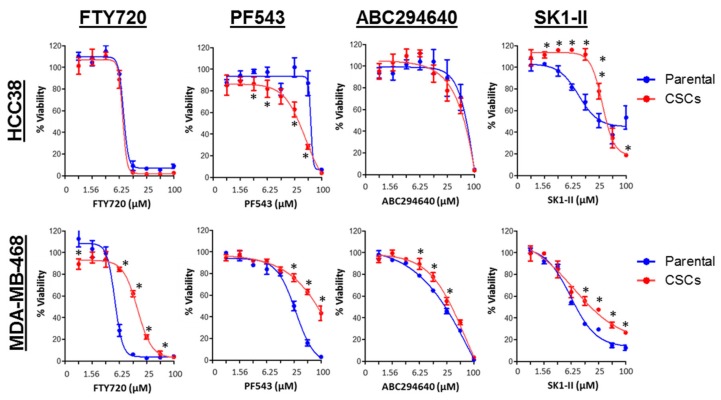
Selective growth inhibitory activities of SPHK inhibitors in parental and breast CSCs. HCC38 and MDA-MB-468 parental and CSCs were treated with different concentrations of FTY720, PF543, ABC294640, and SK1-II for 72 h, and cell viability was determined by the MTT or MTS assay. Points represent the mean ± s.d. of three independent experiments. Asterisks (*) indicate statistical significance compared with parental cells (*p* < 0.05, Student’s *t*-test).

Consistent with previous studies, the breast CSCs were inherently more resistant to doxorubicin compared to the parental cells (two-fold in HCC38 and 14-fold in MDA-MB-468; Table 1) [52,56]. However, we found that the HCC38 CSCs were more sensitive to PF543, but resistant to SK1-II, as compared with the parental cells, while FTY720 and ABC294640 showed equal potency against both the parental and CSCs in HCC38 cells. The high EC_50_ values determined for PF543 suggested that at higher concentrations of PF543, there might be “off-target” effects. In addition, the effect of ABC294640 is unlikely attributable to SPHK2 in CSCs, as it is absent in these cells. In contrast, the MDA-MB-468 CSCs were found to be slightly more resistant (1.4- to our-fold) to all the SPHK inhibitors as compared with the parental cells (Figure 11).

**Figure 12:**
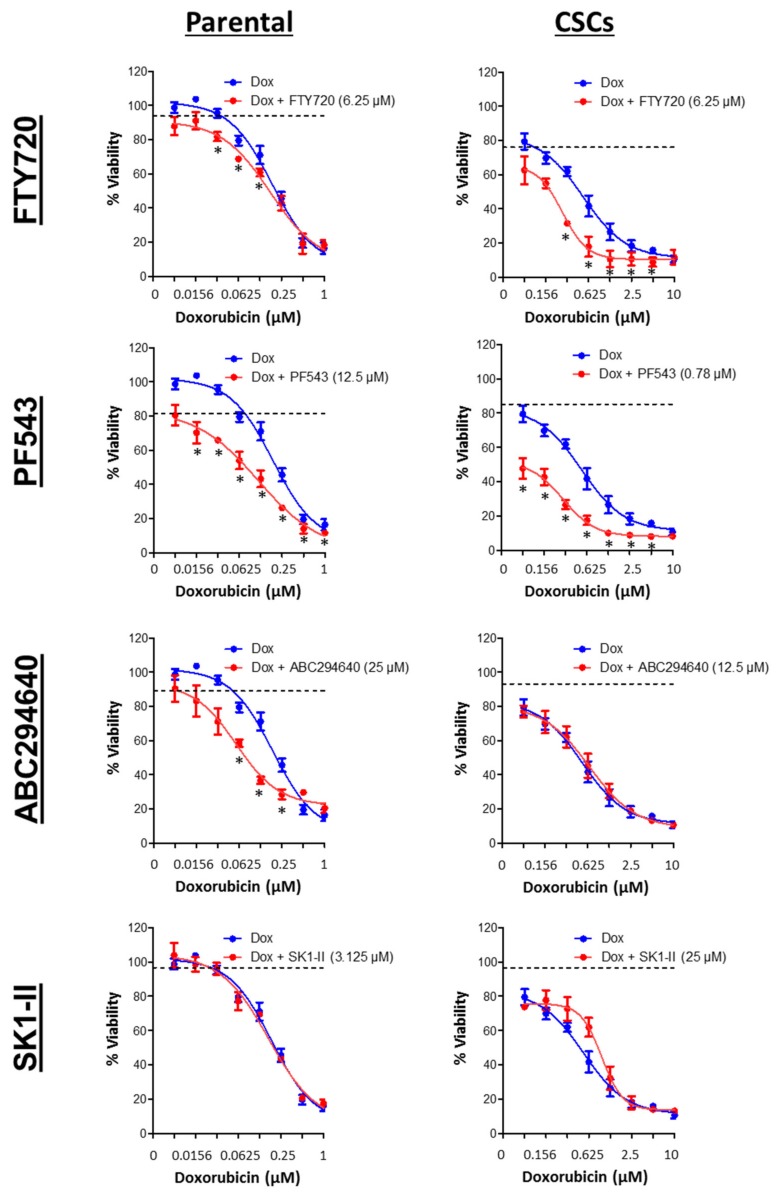
Combination effects of SPHK inhibitors and doxorubicin (Dox) against HCC38 parental and CSCs. Cells were treated with a sublethal concentration of SPHK inhibitors in combination with doxorubicin for 72 h. Cell viability was determined by the MTT or MTS assay. The dashed line represents the cell viability following treatment of the indicated concentrations of FTY720, PF543, ABC294640, and SK1-II. Points represent the mean ± s.d. of three independent experiments. Asterisks (*) indicate statistical significance compared with cells treated with either doxorubicin or SPHK inhibitors alone (*p* < 0.05, Student’s *t*-test).

**Figure 13:**
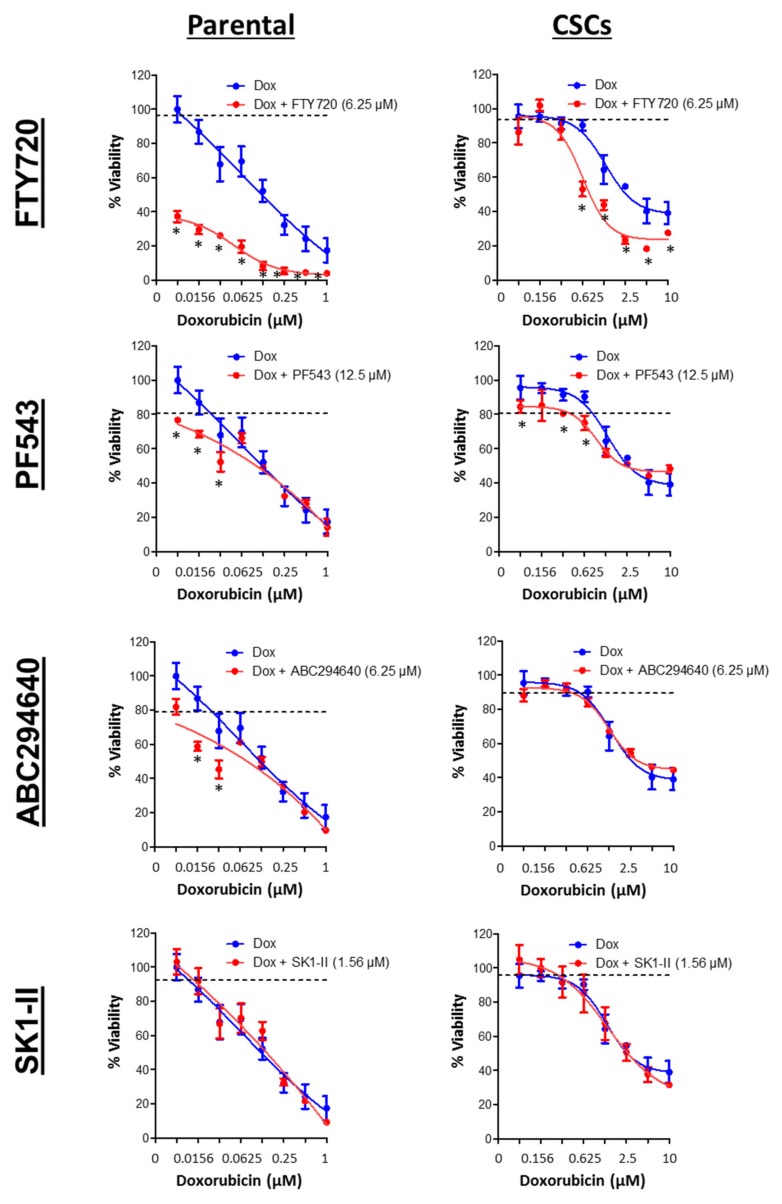
Combination effects of SPHK inhibitors and doxorubicin (Dox) against MDA-MB-468 parental and CSCs. Cells were treated with a sub-lethal concentration of SPHK inhibitors in combination with doxorubicin for 72 h. Cell viability was determined by the MTT or MTS assay. The dashed line represents the cell viability following treatment of the indicated concentrations of FTY720, PF543, ABC294640, and SK1-II. Points represent the mean ± s.d. of three independent experiments. Asterisks (*) indicate statistical significance compared with cells treated with either doxorubicin or SPHK inhibitors alone (*p* < 0.05, Student’s *t*-test).

Next, we investigated whether SPHK inhibitors exhibit synergism with conventional chemotherapy, specifically doxorubicin, in eradicating both breast CSCs and non-CSCs. Our results indicated that combinations of FTY720 or PF543 synergized with doxorubicin in both parental and CSC cultures of basal-like HCC38 and MDA-MB-468 cells. A very low concentrations of PF543 (0.78 μM) was used to induce synergism with doxorubicin in CSC cultures of HCC38 cells, and this is a concentration at which SPHK1 is selectively inhibited. ABC294640 exhibited selective synergism with doxorubicin in the parental cells, but not CSCs of HCC38 and MDA-MB-468, consistent with the lack of expression of SPHK2 in CSCs (Figure 12 and Figure 13). In contrast, the combination of doxorubicin and SK1-II exerted antagonistic effect in both parental and CSC cultures of HCC38 and MDA-MB-468. In this case, it is interesting to note that SK1-II can also induce the polyubiquitination of dihydroceramide desaturase, and these forms are linked with pro-survival p38 MAPK/JNK and XBP-1s pathways in HEK293T cells [76]. 

Taken together, our results suggested that inhibitors targeting SPHK1, but not SPHK2, potentiated doxorubicin-induced cytotoxicity in the basal-like breast CSCs and non-CSCs.

## 4. Discussion

In this study, we demonstrated that the SPHK1-S1P axis is hyper-activated in breast CSCs and promotes cell survival in both breast CSCs and non-CSCs by suppressing STAT1 expression. We further demonstrated that depletion of SPHK1 led to de-repression of STAT1 and induced type I and type II interferon signaling. Importantly, selective inhibition of SPHK1 (but not SPHK2) enhanced doxorubicin sensitivity in breast CSCs and non-CSCs. Collectively our findings suggested a novel role for SPHK1 in attenuating STAT1-mediated IFN signaling, and therapies targeting this signaling axis may be beneficial to breast cancer patients. 

Overexpression of SPHK1 is associated with tumorigenic cell behavior that involves promoting cell growth, migration, and inhibiting apoptosis [24,25,26,38,39,40,41,44]. Recent studies also indicated that SPHK1-S1P may play a role in regulating CSCs populations in breast cancer models. Stimulation of SPHK1/S1P/S1P_3_ by environmental carcinogens promoted CSC-induced tumor metastasis in vivo; while knockdown of S1P_3_ reduced the CSC population in MCF-7 cells [50]. Similarly, the presence of S1P induces Notch signaling and augments mammosphere-forming capacity in breast CSCs, and S1P_3_ is highly expressed in ALDH-positive cells derived from breast cancer patients, suggesting that the SPHK1/S1P/S1P_3_ axis is important for the maintenance of stem-like features [49].

Although the mechanism by which SPHK1 suppresses STAT1 phosphorylation and downregulates total STAT1 levels remains to be further investigated, several pieces of evidence suggest that S1P might play an essential role. Indeed, recent studies have shown that activation of S1P_1_ and S1P_2_ by S1P suppresses IFN and STAT1 activity [77,78]. In addition, S1P binding to S1P_1_ has also been shown to promote STAT3 activation [30,79,80], which is pro-tumorigenic in breast cancer [81]. Thus, a reduction in S1P levels with SPHK1 knockdown might inhibit these pathways and relieve inhibition of the STAT1/IFN pathway.

Important evidence also demonstrates the tumor suppressor properties of STAT1 in mammary tumorigenesis [66,67,68,82,83]. For instance, STAT1 activation has been shown to regulate the expression of cell cycle regulators transcriptionally (upregulates p21WAF1 and p27KIP1 and downregulates cyclins A and E, CDK2). It also enhances the function of pro-apoptotic proteins (upregulates BAK and inhibits transcription of BCL2 and BCL-XL) and death-receptors and their ligands (induces TNF, FAS, TRAIL) [82,83]. Recently, STAT1 has also been shown to modulate cell death by direct protein–protein interaction with p53 following DNA damage [84,85].

Our results indicated that the selective SPHK1 inhibitor, PF543, synergized with doxorubicin in both parental and CSC cultures of basal-like HCC38 and MDA-MB-468 cells, indicating that SPHK1 and its regulation of STAT1 might be of therapeutic utility. Of note, DNA damage and doxorubicin have been previously shown to downregulate SPHK1 protein expression and activity via proteasomal degradation of the enzyme [86,87].

The SPHK2 inhibitor, ABC294640, exhibited selective synergism with doxorubicin in the parental cells, but not CSCs of HCC38 and MDA-MB-468 cells. The role of SPHK2 in cancer is more controversial, with evidence showing both oncogenic and anti-cancer effects. Overexpression of SPHK2 was reported to suppress cell growth and induce apoptosis by sequestration of BCL2 by its BH3 domain [88], while siRNA-mediated loss of SPHK2 from cancer cells produced a strong anti-cancer effects [89]. In addition, the treatment of early stage and advanced prostate cancer cells with the selective SPHK2 inhibitor, ABC294640, induces a reduction in Myc and androgen receptor (AR) expression, and this is associated with significant inhibition of growth, proliferation, and cell cycle progression [90]. Furthermore, knockout of the Sphk2 gene reduces leukemia development in a mouse model of acute lymphoblastic leukemia (ALL), and pharmacologic inhibition extends survival of mice in xenograft models of human disease [76]. SPHK2 is also implicated in multiple myeloma [91] and the SPHK2 inhibitor, (*R*)-FTY720 methyl ether (ROMe), induces the autophagic death of T-ALL cell lines [91].

Despite the important roles of sphingolipid metabolism in cancer biology, only very few agents targeting this pathway have reached clinical trials relating to cancer. These include safingol (L-threo-dihydrosphingosine), a PKC inhibitor (Phase 1; NCT01553071 and NCT00084812); ABC294640, a SPHK2 inhibitor (Phase I/II; NCT01488513, NCT02229981, NCT02757326, NCT02939807, NCT03377179, and NCT03414489); sonepcizumab (ASONEP), an S1P-specific monoclonal antibody (Phase I/II; NCT00661414 and NCT01762033); and Fingolimod, an S1P receptor antagonist (Phase I; NCT02490930). Given the synergistic effects of PF543 and doxorubicin on breast CSCs and non-CSCs in our study, it was proposed that neoadjuvant treatment with SPHK1 inhibitors with doxorubicin would reduce both CSCs and non-CSCs and thereby potentially improve therapeutic response in breast cancer patients. 

## 5. Conclusions

In conclusion, we demonstrated that SPHK1 is required for breast CSCs’ and non-CSCs’ survival through suppression of the tumor suppressor function of STAT1. Targeting SPHK1 using PF543 significantly synergized the anti-proliferative effects of doxorubicin. Hence, targeting SPHK1-S1P-STAT1 could trigger multifaceted anti-tumor responses and may be a promising approach warranting further development. 

## Figures and Tables

**Table 1:** EC_50_ values of doxorubicin and SPHK inhibitors in parental and breast CSCs.

Drugs	HCC38		MDA-MB-468	
	Parental	CSCs	Parental	CSCs
Doxorubicin (µM)	0.20 ± 0.02	0.59 ± 0.14	0.15 ± 0.04	2.22 ± 1.03
FTY720 (µM)	9.50 ± 0.13	9.04 ± 0.33	5.30 ± 0.14	16.29 ± 0.81
PF543 (µM)	72.86 ± 4.68	33.89 ± 3.67	21.55 ± 7.67	85.98 ± 15.70
ABC294640 (µM)	65.20 ± 6.42	60.85 ± 5.10	22.57 ± 1.02	31.56 ± 3.01
SK1-II (µM)	29.70 ± 11.56	41.36 ± 4.73	7.58 ± 0.52	15.72 ± 5.21

## Supplementary Materials

The following are available online at https://www.mdpi.com/2073-4409/9/4/886/s1. Figure S1: mRNA Expression of SPHK1 and SPHK2 in breast CSCs and non-CSCs, Table S1: List of proteins that were upregulated in HEK-293 cells following ectopic expression of SPHK1 (log2 ratio of SPHK1/vector > 2), Table S2: List of proteins that were downregulated in HEK-293 cells following ectopic expression of SPHK1 (log2 ratio of SPHK1/vector < -2), Table S3: List of pathways that were significantly enriched in HEK-293 cells following ectopic expression of SPHK1 (*p* < 0.05), Table S4: shRNA target sequences for SPHK1 and STAT1, and Table S5: Forward and reverse primer sequences for quantitative RT-PCR, Supplemental Methods: Proteomic profiling using LC-MS/MS analysis.
