# Supplementary material for: Sphingosine Kinase 1 Regulates the Survival of Breast Cancer Stem Cells and Non-stem Breast Cancer Cells by Suppression of STAT1
Source: Cells. 2020 Apr 4;9(4):886. doi: 10.3390/cells9040886 (PMC7226795; doi:10.3390/cells9040886)
Supplement: Supplementary file 1 [file cells-09-00886-s001.pdf]

**SUPPLEMENTAL FIGURES**

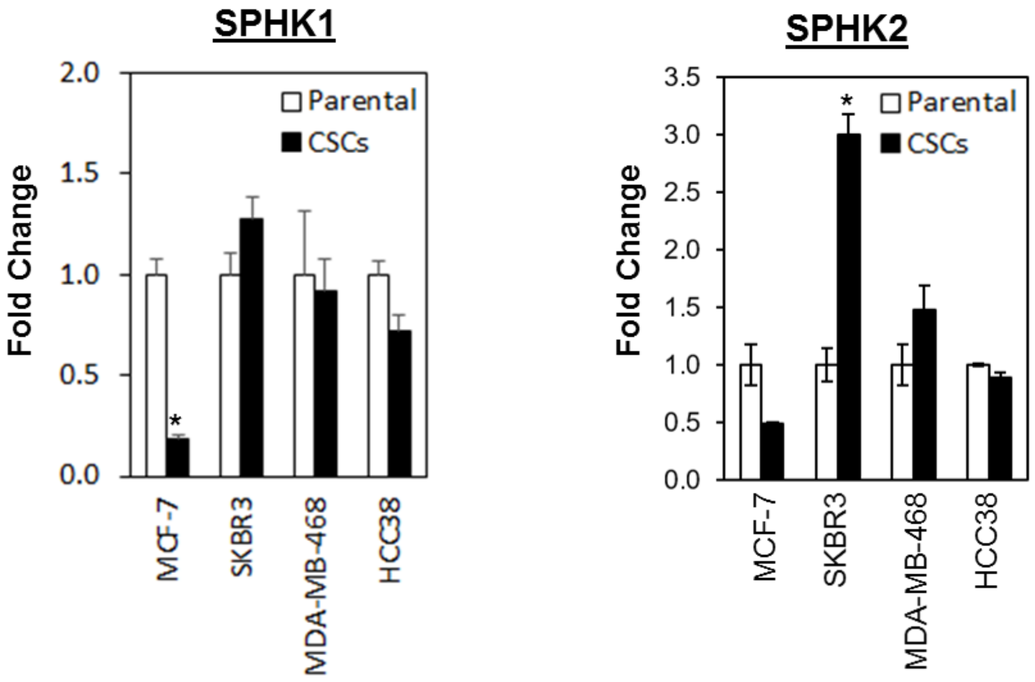


**Figure S1: mRNA Expression of SPHK1 and SPHK2 in breast CSCs and non-CSCs.** mRNA from both parental and CSCs were harvested using RNeasy Mini Kit (Qiagen, USA). High Capacity RNA-to-cDNA Master Mix (Applied Biosystems, USA) was used to prepare cDNA. Gene expression was measured using FastStart Universal SYBP Green Master reagent (Roche, USA) in Bio-Rad iQ5 real-time quantitative real-time polymerase chain reaction (qPCR) detector system (BioRad Laboratories, USA). The forward and reverse primers used in qPCR reactions are shown in Table S5. The following qPCR reactions were applied: 94°C (3 minutes), then a total of 40 cycles of 94°C (40 seconds), 60°C (40 seconds), and 72°C (25 seconds). All qPCR results were normalized using GAPDH, the house keeping gene. Bars represent the means ± s.d. of 3 independent experiments. Asterisks (*) indicate statistical significance compared with non-silencing (NS) control cells ($P < 0.01$, Student's *t*-test).

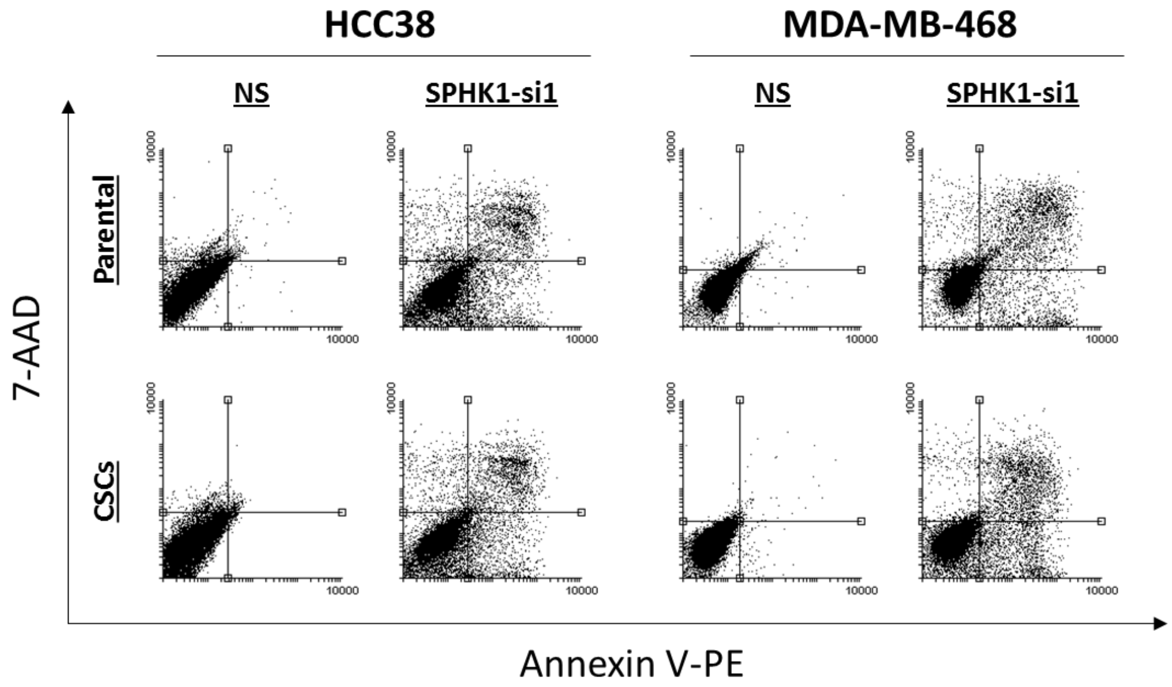


**Figure S2: Induction of apoptosis in breast CSC and non-CSC cultures following depletion of SPHK1.** Representative flow cytometry analysis of cells transduced with either SPHK1-directed shRNA (SPHK1-si1) or a non-specific shRNA control (NS). Unfixed cells were stained with annexin V-PE and 7-aminoactinomycin D (7-AAD) at 72 hours following SPHK1 knock-down, then analyzed by flow cytometry.

**SUPPLEMENTAL TABLES**

**Table S1: List of proteins that were up-regulated in HEK-293 cells following ectopic expression of SPHK1 ($log_2$ ratio of SPHK1/vector > 2).**

| Gene Symbol | Full Name |
|---|---|
| ABL1 | Tyrosine-protein kinase ABL1 |
| AFP | Alpha-fetoprotein |
| AHSG | Alpha-2-HS-glycoprotein;Alpha-2-HS-glycoprotein chain A;Alpha-2-HS-glycoprotein chain B |
| ALB | Serum albumin |
| AMBP | Protein AMBP;Alpha-1-microglobulin;Inter-alpha-trypsin inhibitor light chain;Trypstatin |
| ARHGAP45 | Minor histocompatibility protein HA-1;Minor histocompatibility antigen HA-1 |
| ASNS | Asparagine synthetase [glutamine-hydrolyzing] |
| C11orf96 | Chromosome 11 open reading frame 96 |
| C4B | Complement C4-B;Complement C4 beta chain;Complement C4-B alpha chain;C4a anaphylatoxin;C4b-B;C4d-B;Complement C4 gamma chain |
| CBSL | Cystathionine beta-synthase like |
| CD40 | Tumor necrosis factor receptor superfamily member 5 |
| CFAP100 | Coiled-coil domain-containing protein 37 |
| COMP | Cartilage oligomeric matrix protein |
| CTH | Cystathionine gamma-lyase |
| DCAF13 | DDB1- and CUL4-associated factor 13 |
| DKK3 | Dickkopf-related protein 3 |
| EFEMP1 | EGF-containing fibulin-like extracellular matrix protein 1 |
| EIF4EBP1 | Eukaryotic translation initiation factor 4E-binding protein 1 |
| GC | Vitamin D-binding protein |
| GPX1 | Glutathione peroxidase 1 |
| GPX4 | Phospholipid hydroperoxide glutathione peroxidase, mitochondrial |
| ITIH2 | Inter-alpha-trypsin inhibitor heavy chain H2 |
| KNG1 | Kininogen-1;Kininogen-1 heavy chain;T-kinin;Bradykinin;Lysyl-bradykinin;Kininogen-1 light chain;Low molecular weight growth-promoting factor |
| LGALS13 | Galactoside-binding soluble lectin 13 |

| | |
|---|---|
| PPP4R4 | Serine/threonine-protein phosphatase 4 regulatory subunit 4 |
| PSAT1 | Phosphoserine aminotransferase |
| QPRT | Nicotinate-nucleotide pyrophosphorylase [carboxylating] |
| SELENOF | 15 kDa selenoprotein |
| SELENOH | Selenoprotein H |
| SERPINA1 | Alpha-1-antitrypsin;Short peptide from AAT |
| SERPINF2 | Alpha-2-antiplasmin |
| SLC7A1 | High affinity cationic amino acid transporter 1 |
| SLFNL1 | Schlafen-like protein 1 |
| TXNIP | Thioredoxin-interacting protein |
| ZNF717 | Zinc finger protein 717 |

**Table S2: List of proteins that were down-regulated in HEK-293 cells following ectopic expression of SPHK1 ($log_2$ ratio of SPHK1/vector < -2).**

| Gene Symbol | Full Name |
|---|---|
| ADM | ADM;Adrenomedullin;Proadrenomedullin N-20 terminal peptide |
| APOL2 | Apolipoprotein L2 |
| BRD2 | Bromodomain-containing protein 2 |
| BRD3 | Bromodomain-containing protein 3 |
| SHFL | Shiftless antiviral inhibitor of ribosomal frameshifting |
| CCDC175 | Coiled-coil domain-containing protein 175 |
| CD14 | Monocyte differentiation antigen CD14;Monocyte differentiation antigen CD14, urinary form;Monocyte differentiation antigen CD14, membrane-bound form |
| CD274 | Programmed cell death 1 ligand 1 |
| CLIC3 | Chloride intracellular channel protein 3 |
| CMPK2 | UMP-CMP kinase 2, mitochondrial |
| CTSF | Cathepsin F |
| DDX58 | Probable ATP-dependent RNA helicase DDX58 |
| DHRS2 | Dehydrogenase/reductase SDR family member 2, mitochondrial |
| DTX3L | E3 ubiquitin-protein ligase DTX3L |
| ECHDC3 | Enoyl-CoA hydratase domain-containing protein 3, mitochondrial |

| | |
|---|---|
| EPS8L1 | Epidermal growth factor receptor kinase substrate 8-like protein 1 |
| FADS3 | Fatty acid desaturase 3 |
| GBP1 | Interferon-induced guanylate-binding protein 1 |
| GJB3 | Gap junction beta-3 protein |
| HERC3 | Probable E3 ubiquitin-protein ligase HERC3 |
| HLA-B | HLA class I histocompatibility antigen, B-8 alpha chain |
| HLA-B | HLA class I histocompatibility antigen, B-7 alpha chain |
| HLA-C | HLA class I histocompatibility antigen, Cw-7 alpha chain |
| HLA-C | HLA class I histocompatibility antigen, Cw-1 alpha chain |
| HLA-F | HLA class I histocompatibility antigen, alpha chain F |
| HS3ST1 | Heparan sulfate glucosamine 3-O-sulfotransferase 1 |
| IFI35 | Interferon-induced 35 kDa protein |
| IFI6 | Interferon alpha-inducible protein 6 |
| IFIT1 | Interferon-induced protein with tetratricopeptide repeats 1 |
| IFIT2 | Interferon-induced protein with tetratricopeptide repeats 2 |
| IFIT3 | Interferon-induced protein with tetratricopeptide repeats 3 |
| IFIT5 | Interferon-induced protein with tetratricopeptide repeats 5 |
| IFITM3 | Interferon-induced transmembrane protein 3 |
| IL1RN | Interleukin-1 receptor antagonist protein |
| IRF9 | Interferon regulatory factor 9 |
| ISG15 | Ubiquitin-like protein ISG15 |
| KRT14 | Keratin, type I cytoskeletal 14 |
| KRT17 | Keratin, type I cytoskeletal 17 |
| KRT3 | Keratin, type II cytoskeletal 3 |
| LGALS3BP | Galectin-3-binding protein |
| LY75 | Lymphocyte antigen 75 |
| MGARP | Protein MGARP |
| MGST3 | Microsomal glutathione S-transferase 3 |
| MNDA | Myeloid cell nuclear differentiation antigen |
| MX1 | Interferon-induced GTP-binding protein Mx1;Interferon-induced GTP-binding protein Mx1, N-terminally processed |
| NIP7 | 60S ribosome subunit biogenesis protein NIP7 homolog |
| NMI | N-myc-interactor |

| | |
|---|---|
| NPEPL1 | Probable aminopeptidase NPEPL1 |
| NWD2 | NACHT and WD repeat domain-containing protein 2 |
| OAS1 | 2'-5'-oligoadenylate synthase 1 |
| OAS2 | 2'-5'-oligoadenylate synthase 2 |
| OAS3 | 2'-5'-oligoadenylate synthase 3 |
| PARP12 | Poly [ADP-ribose] polymerase 12 |
| PARP14 | Poly [ADP-ribose] polymerase 14 |
| PARP9 | Poly [ADP-ribose] polymerase 9 |
| PIN4 | Peptidyl-prolyl cis-trans isomerase NIMA-interacting 4 |
| PLEKHA6 | Pleckstrin homology domain-containing family A member 6 |
| PPIG | Peptidyl-prolyl cis-trans isomerase G |
| PSMB9 | Proteasome subunit beta type-9 |
| PYCARD | Apoptosis-associated speck-like protein containing a CARD |
| RUBCN | Run domain Beclin-1 interacting and cysteine-rich containing protein |
| SAA1 | Serum amyloid A-1 protein;Amyloid protein A;Serum amyloid protein A(2-104);Serum amyloid protein A(3-104);Serum amyloid protein A(2-103);Serum amyloid protein A(2-102);Serum amyloid protein A(4-101) |
| SAA2 | Serum amyloid A-2 protein |
| SAMD9 | Sterile alpha motif domain-containing protein 9 |
| SIX4 | Homeobox protein SIX4 |
| SP110 | Sp110 nuclear body protein |
| SRSF4 | Serine/arginine-rich splicing factor 4 |
| SRSF6 | Serine/arginine-rich splicing factor 6 |
| ST14 | Suppressor of tumorigenicity 14 protein |
| STAP2 | Signal-transducing adaptor protein 2 |
| STAT1 | Signal transducer and activator of transcription 1-alpha/beta |
| TLR3 | Toll-like receptor 3 |
| TMEM40 | Transmembrane protein 40 |
| TRIM22 | E3 ubiquitin-protein ligase TRIM22 |
| UBE2L6 | Ubiquitin/ISG15-conjugating enzyme E2 L6 |
| XAF1 | XIAP-associated factor 1 |
| ZRANB2 | Zinc finger Ran-binding domain-containing protein 2 |

**Table S3: List of pathways that were significantly enriched in HEK-293 cells following ectopic expression of SPHK1 ($P < 0.05$).**

| Pathway | P-value |
|---|---|
| Type I interferon signaling pathway | 3.05E-05 |
| Negative regulation of DNA damage response by p53 | 2.37E-03 |
| Response to virus | 3.51E-03 |
| Release of cytochrome c from mitochondria | 7.42E-03 |
| Interferon-gamma-mediated signaling pathway | 7.59E-03 |
| Intracellular protein transport | 1.09E-02 |
| Positive regulation of cell growth | 1.20E-02 |
| Positive regulation of atpase activity | 1.32E-02 |
| Defense response to virus | 1.43E-02 |
| Negative regulation of apoptotic process | 1.47E-02 |
| Single organismal cell-cell adhesion | 1.96E-02 |
| Response to interferon-beta | 4.98E-02 |

**Table S4: shRNA target sequences for SPHK1 and STAT1.**

| | Target sequence |
|---|---|
| **SPHK1-si1** | 5'-CCGGGCAGCTTCCTTGAACCATTATCTCGAGATAATGGTTCAAGGAAGCTGCTTTTTG-3' |
| **SPHK1-si2** | 5′-CCGGGCAGGCATATGGAGTATGAATCTCGAGATTCATACTCCATATGCCTGCTTTTTG-3′ |
| **STAT1-si1** | 5′-CCGGCCCAAAGTATCAGGACGAGAACTCGAGTTCTCGTCCTGATACTTTGGGTTTTT-3′ |

**Table S5: Forward and reverse primer sequences for quantitative RT-PCR.**

| Target Genes | Sequence (5′-3′) | |
|---|---|---|
| | Primer (Forward) | Primer (Reverse) |
| **SPHK1** | CGGCTGCGAAGTTGAGCGAA | GGTGACCTGCTCATAGCCAGC |
| **SPHK2** | CCCAGTGTTGGAGAGCTGAAGG | CGAGGTAGCAGGTCAGGGGT |
| **GAPDH** | GTCTCCTCTGACTTCAACAGCG | ACCACCCTGTTGCTGTAGCCAA |

**SUPPLEMENTAL METHODS**

*Protein extraction*

Total protein was harvested by trichloroacetic acid (TCA) method followed by acetone precipitation. Briefly, cells were washed twice with PBS and proteins precipitated in 6% TCA solution in 100 mM ammonium bicarbonate. The lysates were centrifuged at maximum speed for 10 minutes at 4°C, and the cell pellet washed twice in 100% ice-cold acetone.

*Enzymatic digestion*

Pellets were resuspended in 8M Urea/100mM ammonium bicarbonate, reduced by incubation with 5mM dithiothreitol (DTT) at room temperature for 30 min and then alkylated with 15 mM iodoacetamide for 30 min at room temperature in the dark. Iodoacetamide was quenched with an additional 5mM DTT. Samples were diluted to 2M Urea using 100 mM ammonium bicarbonate prior to digestion with Lysyl endopeptidase (lysC, Promega) for 6 hours at room temperature. Samples were further diluted to 1M Urea and digested overnight with trypsin at 37 ℃. Digests were acidified by the addition of 10% trifluoroacetic acid (TFA) to 0.5% final concentration and the peptides were desalted on 100mg C18 Sep-Pak cartridges (Waters, USA) and dried in a centrifugal evaporator.

*TMT Labeling and peptide fractionation by high pH reverse phase chromatography*

Peptides were TMT-labelled and pre-fractionated in the manner described in Huang et.al (2017, https://www.ncbi.nlm.nih.gov/pmc/articles/PMC5336479/). Briefly, peptides were resuspended in 0.2 M HEPES buffer, pH 8.5. TMT reagents (only 3 channels) were added to samples and incubated at room temperature for 1 hr, quenched by the addition of 5% hydroxylamine for 15 min and immediately acidified with 100% FA. Reaction mixtures were combined in a 1:1:1 ratio and desalted on a StageTip. Following pre-fractionation using high pH reverse phase chromatography, peptides were analyzed by previously described LC-MS3 methods on a Thermo Orbitrap Fusion Tribrid mass spectrometer (Thermo Fisher Scientific) equipped with an Easy nLC-1000 UHPLC (Thermo Fisher Scientific) (Huang et.al (2017, https://www.ncbi.nlm.nih.gov/pmc/articles/PMC5336479/)).

*Data Analysis*

Mass spectra were processed, filtered, and quantified using a SEQUEST-based software pipeline (https://www.ncbi.nlm.nih.gov/pubmed/21183079/). Briefly, peptide-spectrum matches were adjusted to a 1% false discovery rate (FDR). PSM filtering was carried out using a linear discriminant analysis. TMT quantitation was conducted by extracting a signal-to-noise ratio for each TMT channel; PSMs were identified, quantified, and collapsed to a 1% peptide FDR and further collapsed to the protein level using a 1% protein FDR. Signal-to-noise ratios of < 200 were eliminated from the dataset. A filter of at least 2 or more peptides for each protein identification was set before pathway analysis.
